# Gingival recession: its causes and types, and the importance of orthodontic treatment

**DOI:** 10.1590/2177-6709.21.3.018-029.oin

**Published:** 2016

**Authors:** Ana Suzy Jati, Laurindo Zanco Furquim, Alberto Consolaro

**Affiliations:** 1 DDS, Pontifícia Universidade Católica do Paraná (PUC-PR), Curitiba, Paraná, Brazil. Graduate student of Orthodontics and Facial Orthopedics, Dental Press/Unicesumar, Maringá, Paraná, Brazil.; 2 Professor, Universidade Estadual de Maringá (UEM), School of Dentistry, Department of Orthodontics, Maringá, Paraná, Brazil.; 3 Full professor, Universidade de São Paulo (USP), School of Dentistry, Department of Orthodontics, Bauru, São Paulo, Brazil. Professor, Universidade de São Paulo (USP), School of Dentistry, Graduate Program, Ribeirão Preto, São Paulo, Brazil.

**Keywords:** Gingival recession, Gingival retraction, Orthodontic movement

## Abstract

Gingival recession has direct causes and predisposing factors. Orthodontic treatment is able to prevent recession and even contribute to its treatment, with or without periodontal approach, depending on the type and severity of gingival tissue damage. There is no evidence on the fact that orthodontic treatment alone might induce gingival recession, although it might lead the affected teeth (usually mandibular incisors or maxillary canines) to be involved in situations that act as predisposing factors, allowing direct causes to act and, therefore, trigger recession, especially when the buccal bone plate is very thin or presents with dehiscence. Several aspects regarding the relationship between orthodontic treatment and gingival recession have been addressed, and so has the importance of the periosteum to the mechanism of gingival recession formation. Clinical as well as experimental trials on the subject would help to clarify this matter, of which understanding is not very deep in the related literature.

## Resumo

As recessões gengivais têm causas diretas e fatores predisponentes. O tratamento ortodôntico pode prevenir as recessões e, até, contribuir para o seu tratamento, com ou sem uma abordagem por parte do periodontista, dependendo do tipo e da severidade do comprometimento dos tecidos gengivais. Não há evidências de que o tratamento ortodôntico possa induzir, de forma primária, as recessões gengivais, muito embora possa levar os dentes envolvidos (comumente, os incisivos inferiores e os caninos superiores) a situações que ajam como fatores predisponentes para que as causas diretas possam atuar e produzir recessões - em especial, quando deixa-se uma tábua óssea vestibular muito fina ou, até, com deiscência. Vários aspectos da relação entre o tratamento ortodôntico e as recessões gengivais já foram abordados, assim como a importância do periósteo no mecanismo de formação delas. Trabalhos clínicos e experimentais sobre o assunto ajudariam a esclarecer o assunto, que ainda se apresenta muito pouco aprofundado na literatura pertinente.

Gingival recession is represented by atrophic periodontal changes. The term "atrophy" makes reference to all processes of cell lesion characterized by a decrease in volume and cell population of a given organ or tissue, resulting from sublethal cell aggression, such as hypoxia, mechanical compression, local reduced vascularization, among others. Sublethal cell aggression is essentially reversible.

Atrophic cells have a decrease in volume, they eat themselves up and cause their structural components as well as their organelles to be digested. Thus, their level of energy consumption is reduced and they are able to survive within a hostile environment. Once the causal factor is removed, the process ceases and the number and size of cells might be restored to normal levels; however, it all depends on the severity of tissue lesion and the type of tissue involved. 

## GINGIVAL RECESSION: CONCEPTS AND NOMENCLATURE

**Figure 1:**
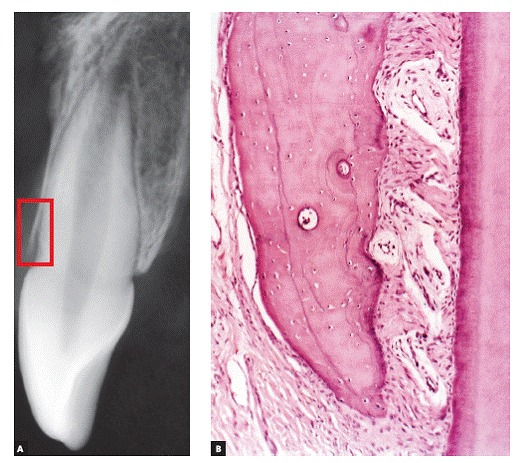
Radiographic image reveals the structural fragility of human buccal bone plate in the region of maxillary incisors, in addition to its dimensional relationship with the periodontal space and the mineralized tooth structures. In B, this proportionality and subtleness are also microscopically revealed (B = 20X HE).

The main characteristic of gingival recession is the apical migration of marginal gingiva as well as the fact that the latter is gradually displaced away from the cementoenamel junction, thereby exposing the root surface to the oral environment (Figs 1 to ^4^). It is found nearly in all populations worldwide and is generally limited to a single root surface - most of times, the buccal one. 

**Figure 2:**
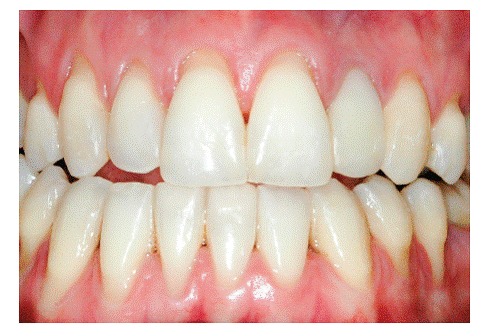
U-shaped gingival recession associated with inadequate tooth brushing, with cervical abrasion of maxillary canines and premolars. Recession is more severe in mandibular premolars.

**Figure 3:**
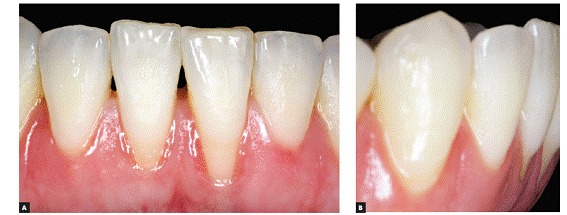
U-shaped local gingival recession associated with reduced alveolar bone plate thickness, a slight increase in dental plaque buildup and inadequate brushing.

**Figure 4:**
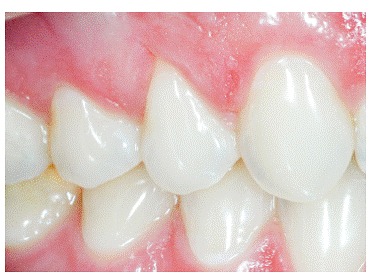
V-shaped gingival recession as a part of occlusal trauma lesion caused by traumatic occlusion.

Within our specialty, the term "gingival recession" is indistinctly used as a synonym for gingival retraction, although some researchers prefer using one rather than the other. In dictionaries, it is possible to find: 1) recession = the act or effect of moving back, withdraw; 2) retraction = the act or effect of retracting; 3) retract = to pull back, to draw back, withdraw. In the present article, we take both terms as synonyms.

For decades, it was believed that gingival recession was a part of human aging processes; however, all evidence supporting such a statement are quite weak. Aging might increase the possibility for the causes of gingival retraction to act, but that does not mean they are inherent to aging. 

Incisal as well as occlusal tooth wear typical of attrition come with aging and are compensated by continuous and passive eruption resulting from uninterrupted cementum deposition in the apical region, which might be sped up. In the event of tooth wear, the cementoenamel junction becomes part of the clinical crown, which might apparently suggest gingival retraction; however, apical gingival migration did not necessarily occur.

**Figure 5:**
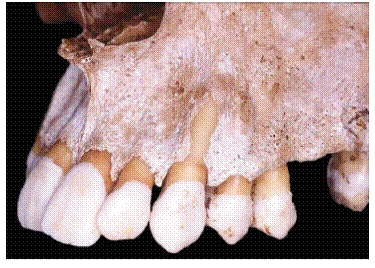
Buccal bone plate with severe canine dehiscence and premolar fenestration.

Gingival recession pathophysiology can be divided into the following: direct causes and predisposing factors. In gingival recession cases, the first mechanism responsible for causing apical gingival migration is loss of bone support offered by the alveolar bone crest (Fig 1). Whenever bone loss is limited to a single tooth surface, usually the buccal one, bone defect is best known as dehiscence (Figs 5, ^6^). Over time, normal or inflamed gingival soft tissues tend to keep up with cervical bone levels; therefore, gingival recession is established.

**Figure 6:**
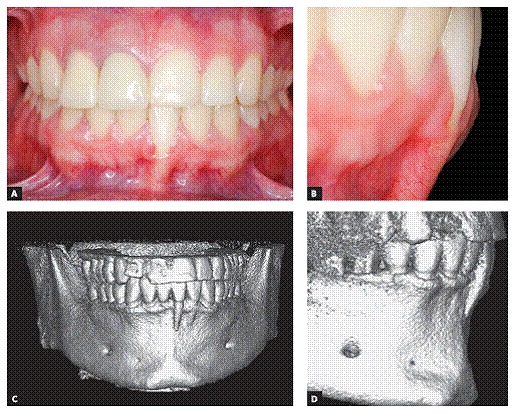
CT scan revealing absence of apical as well as buccal bone plate of a mandibular incisor with gingival recession. Note the presence of inflammation associated with dental plaque and occlusal interference.

## PRIMARY CAUSES OF GINGIVAL RECESSION

***1) Low-level and long-lasting trauma.*** It is also known as chronic trauma, especially due to inappropriate daily brushing, and it physically wounds gingival tissues (Figs 2, ^3^, ^6^). Traumatically using the tooth brush as well as other oral hygiene agents over delicate gingival margins on a daily basis might gradually and slowly lead to gingival recession over the years. In general, those cases are presented in combination with cervical wear as a result of abrasion caused by the same agents. 

***2) Chronic inflammatory periodontal disease.*** Tissue destruction resulting from periodontal disease encompasses gradual bone loss which might lead to apical gingival migration and root exposure. Those cases imply loss of gingival tissue support, as a result of enzymatic digestion and disorganization of the underlying connective tissue, in addition to bone resorption induced by inflammatory process affecting the alveolar bone crest (Figs 2, ^6^). 

At first, tissue loss is apparently compensated by gingival increase resulting from inflammatory exudate and infiltrate accumulation - in other words, by edema, swelling or inflammatory tumefaction. 

After periodontal treatment and once the causes have been eliminated, the exudate will undergo resorption while inflammatory cells will undergo migration, with a decrease and retraction of gingival tissue volume. During the repair process, root will become exposed to the oral environment. Whenever the aforementioned process occurs, the cervical root third becomes exposed which, esthetically, might be considered strange by the patient at the immediate postoperative phase, even though periodontal tissues are perfectly healthy at this point. 

***3) Periodontal treatment.*** Many periodontal treatment modalities imply considerable tissue loss due to extensive periodontal disease or the need for tissue surgical removal. After surgical procedures, such as curettage and surgeries, there is a decrease in periodontal tissue tumefaction, which is temporarily induced by inflammatory exudate accumulation. As repair evolves, there is a decrease in gingival volume and root exposure to the oral environment. Patients themselves are often concerned with treatment outcomes, since gingival inflammation provides gingival enlargement, in addition to covering the cervical region of teeth. 

An interesting measure to be taken is early removal of the majority of agents causing periodontal disease, by means of conservative procedures and before surgical periodontal treatment. This will decrease gingival enlargement caused by inflammatory infiltrate and exudate accumulation, which allows the sites to be subjected to surgery to become better outlined. The patient will notice that root exposure did not result from treatment, but rather from getting rid of the problem.

***4) Occlusal trauma.*** Initially, primary occlusal trauma might induce symptoms characterized by diffuse pain combined with a modest increase in tooth mobility, lasting for days, weeks or even months. 

A few weeks later, an even enlargement of the periodontal space and thickening of lamina dura or alveolar cortical plate becomes radiographically noticeable. These radiographic findings occur as a result of the need for thicker and longer periodontal fibers, so as to give support for the increase in function - in other words, to assimilate the intense occlusal forces. As a result, periodontal ligament thickness increases. Meanwhile, periodontal fibers require an equally greater attachment, which leads to an increase of the alveolar cortical plate, so as to fulfill such a need. This process also applies to the cementum; however, its changes are not revealed by imaging examination.

Also due to the increase in functional demand, caused by excessive occlusal load, there is an intense and continuous stretch of periodontal fibers, especially those attached to the most cervical region of the alveolar bone crest. This overload might cause occasional collagenous fiber structures to break, in addition to over stressing periodontal ligament cells and, as a result, significantly increasing the cervical local levels of chemical mediators released by those cells, especially mediators associated with bone resorption, thereby promoting bone loss, whether vertical or angled, on the periodontal surface of the alveolar bone crest. 

In those cases, vertical bone loss is radiographically noticeable, with the formation of a "V" typical of occlusal trauma, resulting from resorbed bone plane as well as from the root wall. Periapical radiographs can be used for diagnosis, despite interproximal ones being more reliable. No matter how severe vertical bone loss is in this area, periodontal probing will not reveal periodontal pockets. Should occlusal trauma be solved at this stage, periodontal bone neoformation will take place and normality will be restored. All the aforementioned events result from excessive occlusal load within a dental plaque-free environment. For this reason, this set of changes is known as "primary occlusal trauma."

On free surfaces, such as the buccal one, depending on cortical plate or buccal bone plate thickness (Figs 1, ^5^, ^7^, ^8^), vertical bone loss results in local loss at the buccal bone level. This leads to bone dehiscence over the affected root - a V-shaped cavity in the bone contour (Fig 5) -, thereby locally causing gingival bone support to decrease. For a certain period of time, the periosteum might still cover the area affected by dehiscence; thus, favoring bone neoformation if the cause has already been eliminated.

**Figure 7:**
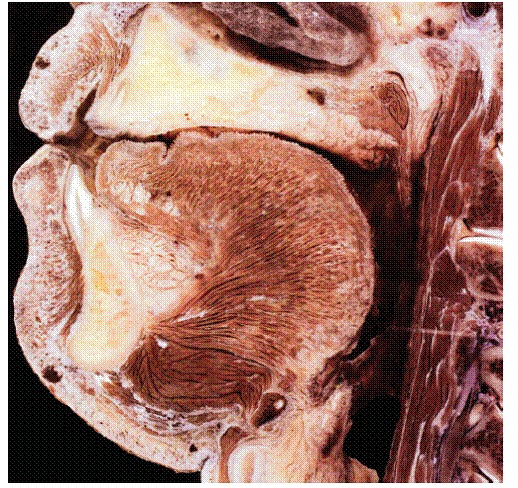
Anatomical piece highlighting buccal bone plate fragility in the region of mandibular incisors (Source: Sgrott, Moreira, 2010 ^15)^.

**Figure 8:**
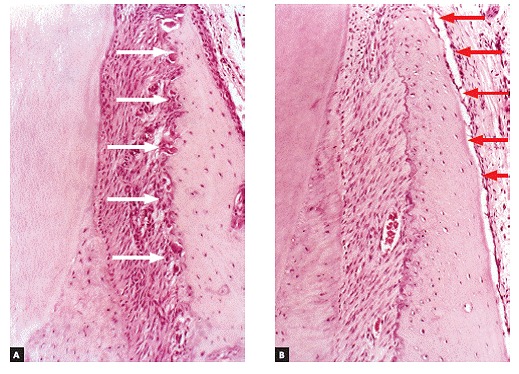
Under orthodontic movement conditions, with balanced load distribution, frontal bone resorption (white arrows) is compensated by deposition of new bone layers on the buccal surface of the buccal bone plate by the periosteum (red arrows) (A and B = 20X, HE).

**Figure 9:**
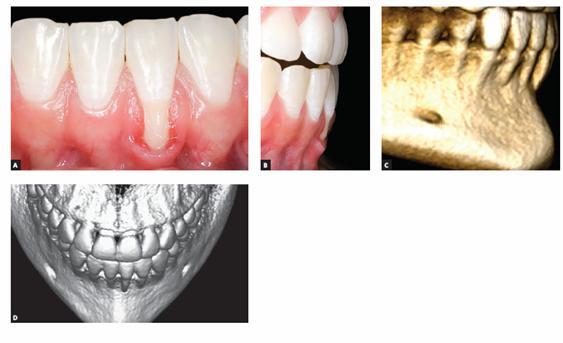
CT scan revealing absence of apical as well as buccal bone plate of a mandibular incisor with gingival recession. Note the red festoon surrounding the recession, which suggests the presence of inflammation associated with dental plaque.

**Figure 10:**
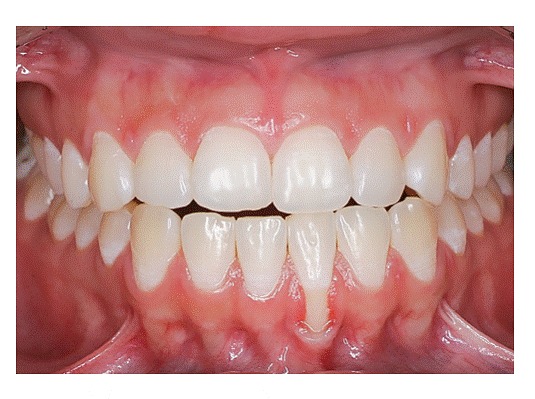
In the same clinical case presented in Figure 9, local gingival recession is associated with dental plaque and severe occlusal interference.

**Figure 11:**
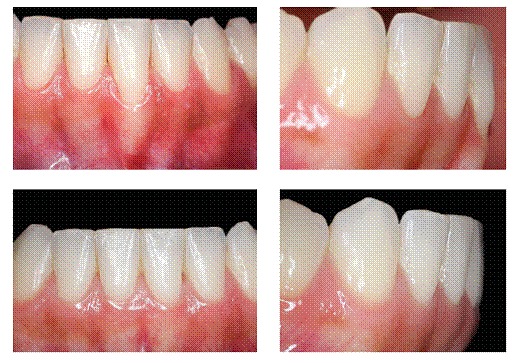
U-shaped local recession corrected by orthodontic repositioning of the mandibular incisor, which allowed the buccal bone plate to become thicker and the exposed root area to be covered by gingiva.

Gingival contour will keep up with buccal bone contour, which results in V-shaped or angled gingival recession in teeth affected by occlusal trauma and bone dehiscence (Fig 4). It should be once again highlighted that this process is not associated with local dental plaque buildup and consequent chronic inflammatory periodontal disease.

Clinically, occlusal or incisal wear, as well as the presence of V-shaped recession, is noticeable. A third sign can be added: abfraction, with cervical enamel cracks or enamel linear loss. In addition, increased sensitivity might also be present due to a number of oral factors, such as eating, liquid intake, breathing, temperature, among others. Due to being a subclinical condition, occlusal trauma might silently evolve to more severe consequences, including root resorption, over time.

Unfortunately, not all professionals are able to make an accurate diagnosis of abfraction and V-shaped gingival recession as clinical manifestations of occlusal trauma. The same applies to imaging changes resulting from those conditions over time. Once occlusion has been corrected, it is possible to repair the damage by means of: restoration of surfaces with wear facets; restorative correction of abfraction, or, should V-shaped gingival recession be too severe, gingival graft might be used. Nevertheless, sometimes gingival recession recedes without surgery after occlusal trauma has been eliminated (Figs 9, ^10^, ^11^). Chronic occlusal trauma treatment might be carried out by tooth repositioning through orthodontic treatment (Fig 11).

## PREDISPOSING FACTORS OF GINGIVAL RECESSION

***1) Decreased alveolar bone crest thickness,*** combined with delicate gingival margin, commonly found in maxillary canines and mandibular incisors (Fig 7). Inappropriate tooth brushing and dental plaque buildup might lead to early gingival recession (Figs 2, ^3^, ^6^). Delicate alveolar cortical plates are not revealed by CT scans nor reproduced by 3D reconstruction (Figs 6, ^9^).

***2) Dehiscence.*** Reduced buccal bone crest thickness might be associated with areas where buccal bone plate is absent - which characterizes dehiscence, when a depression is located apically to the alveolar bone cervical contour; or fenestration, when there is a bone window on the buccal surface. 

With dehiscence and fenestration, the chances of gingival recession occurring are much higher. Those bone crest morphological defects are predisposing factors of gingival recession and are more frequently found in teeth malpositioned in the dental arch, especially uprighted teeth, as it is the case of canines subjected to orthodontic traction and which had erupted more uprightedly. 

***3) Frenulum insertion*** near the cervical region of gingiva. Moving labial and lingual frenula as well as cicatricial adhesions might predispose the region to gingival retraction, especially in the areas subjected to inadequate brushing associated with chronic periodontal disease. 

## DOES ORTHODONTIC TREATMENT INDUCE GINGIVAL RECESSION? FIRST, DEHISCENCE! HOW CAN WE AVOID IT?

Induced tooth movement does not cause any damage to gingival tissues; however, during orthodontic treatment, the following might occur in a few patients: retraction on the buccal surface of incisors and canines, or even in posterior teeth when in combination with lateral movement. Nevertheless, in those cases, before gingival retraction occurs, orthodontic movement had induced dehiscence at the bone crest, as a result of moving a tooth towards an area with extremely thin bone (Fig 8). 

Induced tooth movement should be carried out only at the alveolar bone trabeculae space; however, during certain types of movement, teeth are also displaced at the expenses of the outer cortical plate. Should that be the case, dehiscence and fenestration are established. The latter are defects found in the outer cortical plate and act as "predisposing factors" of gingival retraction. The ideal would be that teeth remain duly "enveloped" by bone tissue in all of their surfaces. Movement should be carefully planned and include more than only one tooth. It should also consider a homogeneous load distribution which favors the compensating bone neoformation mechanism on the corresponding outer periosteal surface. However, this is not always taken into account during treatment planning.^17^ ^,^ ^19^

Orthodontic tooth movement should not be considered as the primary cause of gingival retraction.^3^ ^,^ ^8^ ^,^ ^9^ ^,^ ^14^ ^,^ ^18^ Whenever the latter is present, it is a consequence of changes in periodontal bone morphology: the more delicate the cortical plate and marginal gingiva, the greater the likelihood of gingival retraction occurring as a result of mechanical action exerted by brushing and/or dental plaque buildup.^1^

A potential means to avoid dehiscence and recession during orthodontic treatment is to apply light, well-balanced forces to sets of teeth rather than to a single tooth. Local periosteum receives stimuli from deformed mediators, so as to have new overlying layers laid, thereby covering and causing the buccal cortical plate to become thicker as the teeth are buccally displaced, which compensates for frontal resorption at the periodontal wall of the alveolar cortical plate (Fig 8). 

Orthodontic movement not only affects periodontal tissue volume and shape, but deflection also deforms the alveolar bone process network of osteocytes, which controls bone shape and volume according to functional demand. Buccal bone deflection as a whole provides periosteal stimuli, so as to have new buccal cortical plate layers laid. Whenever movement of individual teeth is rendered necessary, light forces should be applied and body movement carried out, so as to allow the same compensating periosteal mechanism to act. In other words, whatever undergoes resorption at the periodontal surface of the alveolar bone ends up being laid at the corresponding outer buccal surface.

## CLINICAL ASPECTS OF GINGIVAL RECESSION 

Gingival recession might be present in some teeth separately; however, whenever it is generally present, it often affects a whole segment in the dental arch, thus horizontally retracting periodontal tissue attachment, including gingival papillae. As for shape and distribution, they might as well be:

### 1) Local gingival retraction

Local gingival retraction might be V- or U-shaped. 

1.1) ***V-shaped local retraction*** is associated with teeth subjected to occlusal trauma, especially in patients with bruxism and clenching habits (Fig 4). In cases of severe apical migration, V-shaped recession is known as " *Stillman's cleft* ." At the corresponding enamel, it is common to find abfraction; while on the occlusal surface, wear facets caused by attrition are commonly found, as being part of a lesion caused by occlusal trauma.

1.2) ***U-shaped local recession*** is generally associated with chronic inflammatory periodontal disease, inadequate tooth brushing or inadequate frenulum insertion (Figs 2, ^3^, ^9^, ^11^).

U-shaped gingival recession associated with inadequate traumatic brushing is surrounded by healthy gingiva and is usually associated with abrasion, with a smooth, polished surface. There are cases of U-shaped retraction in which the area of root exposure is surrounded by a peripheral festoon made up of swollen, inflamed gingival tissue resulting from local dental plaque buildup. A few classical studies found in Periodontology literature refer to the aforementioned condition as " *McCall's festoon.* "

### 2) Generalized or horizontal retraction

In its generalized or horizontal form, gingival retraction is associated with chronic inflammatory destructive periodontal disease. Loss of periodontal support in proximal areas results in compensatory remodeling on the buccal and lingual surfaces, leading to apical displacement of marginal gingiva, including interdental papillae.

## THE PERIOSTEUM IN DEHISCENCE AND GINGIVAL RECESSION

The periosteum is firmly inserted into the surface of cortical bone through Sharpey's fibers that, in turn, are inserted into bone matrix, predominantly made up of collagen (Figs 1, ^8^). The periosteum connective tissue^16^ is divided into two different contiguous layers:

1) The outer layer is mainly formed by fibroblasts. However, it is predominantly fibrous and aims at providing protection to the surface. This layer originates collagen fibers responsible for periosteum insertion into the subjacent cortical bone. 

2) The inner layer of the periosteum is directly related to the cortical bone surface and is characterized for being rich in osteoblasts, preosteoblasts, osteoprogenitor cells, tissue stem cells, clasts and other cells in smaller numbers. 

3) The center of the periosteal structure is intensely vascularized as a result of a network formed by small vessels and which branches off towards the bone surface. This intermediate zone formed by numerous capillaries could represent a third layer that differs in terms of thickness from the periosteum.^16^

From the periosteum all blood nutrition of bone structure is established. The surgical flap of the periosteum is inevitably a traumatic procedure that implies in loss of biological feasibility of the cortical bone surface layer. The osteocytes of the surface layer die and the bone matrix layer that hosted them undergoes resorption - with or without compensatory bone neoformation, depending on local conditions.

The most important indicator of bone vitality and feasibility is the presence of osteocytes within bone lacunae or osteoplasts. Without them, the bone is likely to undergo resorption and to be repositioned posteriorly. When periodontal surgery is performed on free cortical surfaces with thin alveolar bone cortical bone, the split thickness flap technique causes the periosteum to adhere to the cortical bone, thus avoiding surface resorption and, as a consequence, preventing post-surgical bone dehiscence and fenestration. 

## DOES THE PERIOSTEUM REMAIN WITHOUT UNDERLYING CORTICAL PLATE?

In many cases, the buccal surface of incisors and canines, especially the mandibular ones, is so thin that one has the clear impression of mineralized bone being non existing at palpation. The periosteum might be, and usually is, present; with a delicate, thin, underlying bone plate which is little mineralized, thus characterizing a cortical plate that plays the role of the outer bone plate. 

In a few studies, the buccal alveolar cortical plate is unperceivable by CT scans, leading the examiner to believe that the examined region has no supporting periodontal structure.^2^ ^,^ ^4^ ^,^ ^5^However, the latter exists, although it does not reveal as a precise, topographically perceptible image^12^ that could be seen by microscopic slices.

Similarly, in 3D tomographic reconstruction of the anterior region, one might have the wrong impression that incisors are lacking structure and buccal bone organization. In the majority of 3D reconstruction cases, an irregular granulated surface is found in incisors roots, thus suggesting root surface irregularity. It is likely that such an imaging irregularity suggests the presence of periosteum and the thin, delicate buccal bone plate.

In other words, it is difficult to determine the limits of bone dehiscence and fenestration precisely when the bone plate presents with a thin, delicate structure. Likewise, it is also difficult to establish the limits of cervical bone precisely.

In many procedures performed on maxillary bones with a view to isolating the mineralized portion and teeth from soft tissues, when the periosteum adhered to the alveolar process is removed, the thin layer of mineralized tissue, which is strongly associated with and placed between the periosteum and the periodontal ligament of the area, is also removed. Future analyses will give the impression that many dehiscences and fenestrations are present; however, this is not true, since they originated from the preparation of anatomical pieces. 

## ORTHODONTIC MOVEMENT CAN PREVENT GINGIVAL RECESSION IN AREAS OF RISK

Once buccal periodontal fragility has been identified and confirmed by means of imaging examination, in which it might be invisible due to structural fragility, tooth movement plan can be prepared, so as to position the root structure towards the center of the bone.^10^ ^,^ ^11^ As a result, the buccal bone plate will become thicker and perhaps visible by CT scans (Fig 11). 

Periodontal tissue structure and organization will remain normal, but more resistant to mechanical action resulting from inadequate brushing, dental plaque buildup and occasional occlusal interference, whether resulting from bruxism and clenching or not.

Alveolar cortical bones and areas of tendon insertion are the only areas in the human skeleton lacking periosteum. In alveolar cortical bones, the periodontal ligament accounts for and plays the role of the periosteum. In the alveolar bone crest, the periodontal ligament as well as the periosteum are continuous, without structural interruption (Figs 1, ^8^).

## SHOULD GINGIVAL RECESSION BE PRESENT, ORTHODONTIC MOVEMENT WILL IMPROVE IT, BUT IT WILL NOT SOLVE THE PROBLEM

When root exposure has already been present for a few weeks due to recession, root cementum will have been eliminated and periosteum will have been withdrawn apically with the bone plate. The root surface exposed to the oral environment is now full of bacterial lipopolysaccharides (LPS) which, thanks to high toxicity levels, do not allow further recolonization by cementoblasts and reinsertion of periodontal fibers. 

Even if this tooth is orthodontically moved to a more lingual position, gingival and periosteal cervical levels cannot be restored. The periosteum present in the most apical area of the bone crest naturally remains with the periodontal ligament which, in the tooth socket, plays the role of the periosteum. 

Should that be the case, a periodontist is necessary in order to surgically promote gingival tissue reposition.^7,13^ In post-surgical periodontal tissue repair, reposition of cervical levels is achieved at the expenses of a long junctional epithelium of which cells tolerate the previously exposed root surface to have a certain degree of chemical contamination caused by LPS. 

This long junctional epithelium restores clinical and functional normality at the site; however, it is not possible to say that a new ligament and outer bone plate formed at the previously exposed site. The prognosis at the site will be good under adequate hygiene and brushing conditions.

In order to avoid gingival recession, orthodontic intervention must be as early as possible, so as to prevent root surface contamination caused by microbial biofilm buildup and its lipopolysaccharides. 

## FINAL CONSIDERATIONS

Orthodontic movement is able to position the teeth towards the center of the bone, in addition to increasing the structural thickness of buccal periodontal tissues.^6^ Taking periodontal buccal structure as well as bone structure fragility into account and as a predisposing factor for gingival recession, it is possible to assert that adequate orthodontic planning, tailored to suit certain regions of thin buccal bone plates, is capable of preventing it.

Orthodontic treatment alone will rarely promote gingival recession which, in general, has as primary causes some of its direct causes. Orthodontic treatment carried out without any concern about gingival recession triggers one of the most important predisposing factors for the latter, which is represented by the thin, delicate structure found in the buccal, outer bone plate - which is sometimes unperceivable by CT scans.

Orthodontic movement alone might not completely solve cases of previously established gingival recession which require periodontal approach to be carried out. In cases of V-shaped local gingival recession, which is often associated with occlusal trauma, orthodontically correcting the interference and the traumatic occlusion might cause the process to recede without surgical intervention at the site.
